# Safety, Efficacy and Mid-Term Outcome for Transarterial Embolization (TAE) of Renal Angiomyolipoma (AML) Using Ethylene Vinyl Alcohol Copolymer Liquid Embolic Agent (EVOH)

**DOI:** 10.3390/jcm12103385

**Published:** 2023-05-10

**Authors:** Rémi Rolland, Antoine Loubet, Sébastien Bommart, Valérie Monnin-Bares, Hamid Zarqane, Juliette Vanoverschelde, Fanchon Herman, Nicolas Molinari, Hélène Kovacsik

**Affiliations:** 1 Department of Radiology, CHU of Montpellier, Arnaud de Villeneuve Hospital, 34090 Montpellier, France; 2 Department of Medical Statistics and Epidemiology, Centre Hospitalier Universitaire Montpellier, University of Montpellier, 34090 Montpellier, France

**Keywords:** angiomyolipoma, renal, ONYX, outcome, Transcatheter embolization, ethylene vinyl alcohol (EVOH) copolymer, hemorrhage

## Abstract

Transarterial embolization (TAE) of renal angiomyolipoma (AML) is effective in treating and preventing hemorrhage. We report our experience using EVOH with a single-center retrospective study of all AML embolized with EVOH between June 2013 and March 2022 at the Montpellier University Hospital. A total of 29 embolizations were carried out in 24 consecutive patients (mean age: 53.86 years; 21 women and 3 men) with 25 AMLs for severe bleeding, symptomatic AML, tumor size > 4 cm, or presence of aneurysm(s) > 5 mm. Data collected included imaging and clinical outcomes, tuberous sclerosis complex status, change in AML volume, rebleeding, renal function, volume and concentration of EVOH used, and complications. Out of 29 embolizations performed for 25 AMLs, four were performed in an emergency. Technical success was achieved for 24/25 AMLs. Mean AML volume reduction was 53.59% after a mean follow-up time of 446 days using MRI or CT scan. Aneurysms on angiogram and the symptomatological nature of AML, as well as secondary TAE and multiple arterial pedicles, were statistically associated (*p* < 0.05). Two patients (8%) underwent nephrectomy after TAE. Four patients had a second embolization. Minor and major complication rates were 12% and 8%, respectively. Neither rebleeding nor renal function impairment was noticed. TAE of AML using EVOH is, thus, highly effective and safe.

## 1. Introduction

Renal Angiomyolipoma (AML) is the most frequent renal neoplasm, with a 0.4% incidence in the general population [1]. Most AMLs are asymptomatic and discovered fortuitously. However, as the tumor grows, intratumoral blood flow increases, inducing vessel distension and aneurysm formation [2]. The natural course of the disease is revealed by bulking-effect complications and is stated as the most common cause of renal hemorrhage [3], with spontaneous bleeding into the retroperitoneum, which can be life-threatening, as emphasized by recent guidelines [4]. Strongly associated risk factors for severe bleeding are a tumor size > 4 cm [5], intra-tumoral aneurysms > 5 mm [2], and tuberous sclerosis complex [2,3]. The decision for prophylactic treatment can be discussed when those risk factors are present [6]. Treatment options include open surgery, exemplified by enucleation [7], or partial/total nephrectomy [8]. However, other modes of minimally invasive surgical therapy (laparoscopic [9] or robotic partial nephrectomy [10]), percutaneous ablation techniques (radiofrequency ablation [11], cryoablation [12]), or transarterial embolization (TAE) [3], have recently gained popularity as preferential treatments for AML. Indeed, the most relevant AML treatment is guided by sparing normal renal tissue [13]. Therefore, TAE is particularly recommended in this case with high efficacy and low complication rate [3] by targeting the tumor vasculature, which is responsible for the risk of bleeding [14,15].

To this day, numerous agents can be used for the embolization of AML [16], and no study has demonstrated the superiority of one embolic agent over another with regard to treating symptoms, actively bleeding AML, or preventing hemorrhage. Liquid embolic agents allow a complete vascular filling from proximal trunks to the capillary bed, as well as aneurysms. N-butyl cyanoacrylate glue has proven to be very effective in this indication, with a high hemostasis effect, but its vascular penetration can be unpredictable and requires substantial experience (high learning curve) to avoid technical complications [6].

Ethylene vinyl alcohol copolymer (EVOH or Onyx, Micro Therapeutics, Inc., Irvine, CA, USA) is an elastic polymer used as a “magma-like” [17] liquid embolic agent, known for its controlled delivery injection, slow polymerization, non-adhesive nature, high fluoroscopic visibility [18], high vascular penetration, small inflammatory effect on the endothelium [19] and high hemostasis effect [20]. Indeed, its particular characteristics seem to make it a suitable candidate for the embolization of AML to fill their dysplastic vasculature and, thus, control their hemorrhagic risk. Subsequently, the aim of this retrospective study is to assess the safety and efficacy of AML embolization using EVOH, with 29 arterial embolizations for 25 consecutive AMLs in 24 patients.

## 2. Materials and Methods

### 2.1. Patients Demographics and Study Design

We carried out a retrospective observational study of consecutive patients with renal AML who underwent EVOH embolization at the Montpellier University Hospital over a 9-year period between June 2013 and March 2022. We identified and reviewed 24 patients who underwent 29 embolization procedures for 25 AMLs, by searching our clinical database using the indexing terms “angiomyolipoma” and “Onyx”. Our ethics committee approved the study (IRB ID: 202201088) and waived the requirement for informed patient consent in compliance with French legislation on retrospective studies of anonymized data. All medical records and outpatient charts were reviewed. Data collected included patient demographic characteristics, imaging, and clinical outcomes, change in AML volume, volume and concentration of EVOH used, rebleeding, renal function, and complications.

### 2.2. Diagnosis and Tumor Measurements

**Figure 1:**
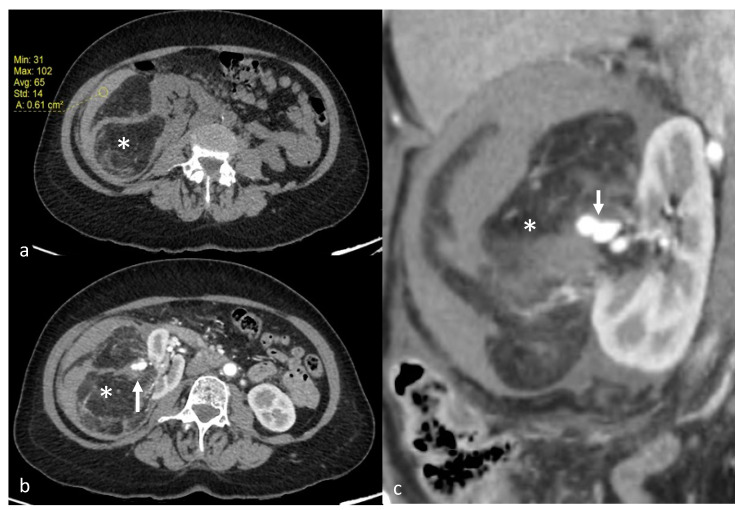
A 70-year-old patient with right AML (*), Wunderlich Syndrome and aneurysm (white arrow). (**a**) Yellow ROI showing retroperitoneal hemorrhage (65 UH) on axial computed tomography without injection; (**b**) Axial computed tomography with injection and arterial acquisition; (**c**) Coronal computed tomography with injection and arterial acquisition.

AML were diagnosed on the basis of macroscopic fat content and the absence of calcification or necrosis at computed tomography (CT; <−20 Hounsfield Units, HU) or magnetic resonance imaging (MRI; cancellation of a high-intensity signal on T1-weighted MR images with fat saturation) [6], before treatment (Figure 1). In the absence of macroscopic fat, the use of in-phase/opposed-phase sequences, low T2W and/or ADC signal, and avid early enhancement with wash-out kinetics with or without intratumoral microscopic fat on chemical shift imaging was used for diagnosis of renal AML without visible fat [21]. In doubtful cases, a biopsy was performed (1 case). To determine the volume of AML, we used three orthogonal diameters (d1, d2, d3) measurements recorded on radiologic investigation (CT or MRI) before embolization and on the most recent CT or MRI obtained during follow-up after embolization. The AML volume was considered ellipsoid, and we used the formula as follows: (d1 × d2 × d3 × π/6) [22]. Aneurysms were diagnosed and measured on diagnostic imaging studies performed with injected contrast material (Figure 1). Patients were referred to an interventional radiologist for embolization after clinical evaluation by a urologist. Indications for TAE were an AML size threshold of greater than 40 mm maximal diameter, an aneurysm of 5 mm or greater or acute hemorrhage from an AML of any size.

### 2.3. Embolization Technique

All TAE procedures were performed by four experienced interventional radiologists who were familiar with the procedure. Three patients had previous embolization with micro-particles and/or micro-coils. These patients were previously embolized in centers that did not use EVOH or radiologists in our center who were not familiar with the use of ONYX.

**Figure 2:**
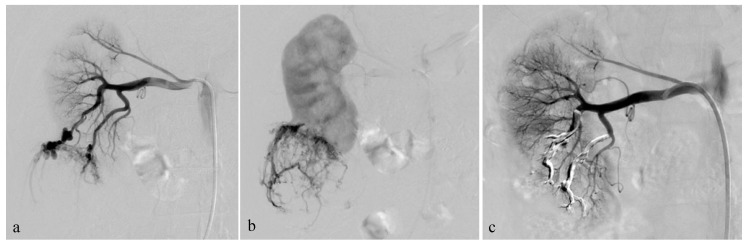
Selective arterial embolization (TAE) of a large right AML. (**a**) Angiography shows two feeding arteries to the AML with multiples aneurysms. (**b**) The hypervascularity and dysplastic vessels of this AML are typical. (**c**) Final control after TAE of two arterial branches with ONYX 18 (6% EVOH) with technical success (lack of opacification of the AML).

The procedures were generally performed under general anesthesia or hypnosedation at the anesthesiologist’s discretion. Systemic anticoagulation was achieved during the procedures with intraarterial heparin in 3000-IU bolus injection, except during emergency procedures. TAE was planned during hospitalization for minimum 24 h. Before undergoing embolization, patients received 1 g of cefazolin (Ancef; GlaxoSmithKline, Philadelphia, Pennsylvania) to prevent superinfection of renal infarction associated with embolization. The right or left common femoral artery was punctured under ultrasound imaging, and a 6-French (Fr) sheath was introduced by Seldinger technique. An aortogram was obtained first through a 6-Fr guiding sheath to locate the renal arteries and to identify any accessory renal arteries or extra-renal feeding arteries. Selective renal angiography was then performed to assess the vascularization of the AML (Figure 2a,b), extension of tumor vessels outside the normal nephrogram, and vessel displacements by the tumor, as well as to identify aneurysms (Figure 2a). In patients with severe bleeding, active extravasation, and retroperitoneal blood were identified. Then, superselective catheterization of the AML feeding vessels was achieved to spare as much renal parenchyma as possible, using a coaxial 2.0 microcatheter with dual marker bands (Echelon^®^; Micro Therapeutics, Inc.^®^ 2005), as well as an Onyx-compatible microcatheter. The dead space of the microcatheter was meticulously flushed with dimethyl sulfoxide (DMSO) solvent to avoid early polymerization of the Onyx in the lumen of the catheter. Onyx (6% or 8% EVOH) was injected at a steady rate of 0.16 mL/min firstly, whilst the dead space was being filled, to avoid development of vasospasm due to DMSO [18]. The EVOH concentration (6% or 8%) was arbitrarily chosen based on our experience with tumor devascularization, considering that the lower the concentration of the copolymer, the less viscous the agent is, allowing a more distal embolization. A total of 6% EVOH was preferably used (88% of AML). If check angiography identified other feeding vessels, further superselective catheterization and embolization were performed. The injection was continued and repeated until the distal end of the feeding arteries of the AML and their dysplastic portions were completely occluded. The endpoint of the procedure was complete devascularization of the tumor (Figure 2c), except when small branches cannot be catheterized (vessel diameter under 1 mm), which was specific to the individual vascular anatomy and clinical situation.

**Figure 3:**
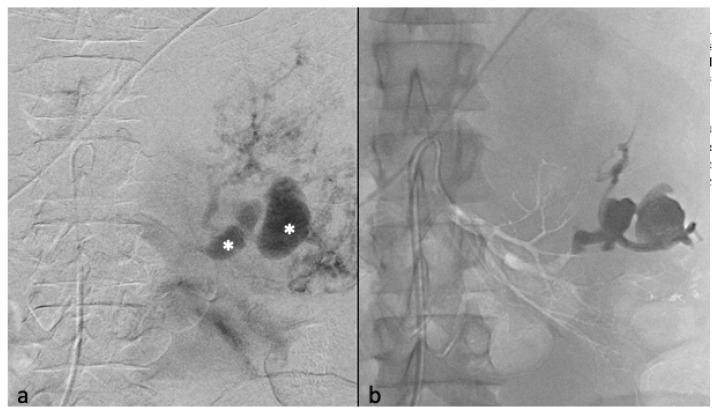
**Transarterial embolization (TAE) of an AML with massive aneurysms.** (**a**) Angiography shows one AML on the upper pole of the left kidney with massive aneurysms (*) (**b**) Final control after TAE of the main arterial branch with ONYX 18 (6% EVOH) and technical success (lack of opacification of the AML).

Postembolization angiography performed through the 6-F catheter confirmed successful occlusion of the artery and the patency of other renal arteries branches. Technical success was defined as no opacification of the main feeding arteries and lack of AML staining on the post-TAE angiography after one embolization (primary technical success) or two (secondary technical success) (Figure 3).

After completion of the injection, the microcatheter was removed by pulling gently during slight aspiration.

### 2.4. Postprocedure Management

Patients were monitored in the interventional unit for the first 2 h and hospitalized for a day. After 24 h, before discharging patients, a Doppler ultrasound of the punctured femoral artery was performed to verify the absence of pseudoaneurysm. A CT scan was also realized to assess the extent of renal infarction associated with embolization. Depending on the extent of renal infarction, a prescription was systematically made for 3 days to 1 week of a nonsteroidal anti-inflammatory drug combined with a proton-pump inhibitor and an analgesic to reduce the clinical manifestations of post-embolization syndrome. Renal function was tested pre-procedure and post-procedure by measuring serum creatinine and estimating the glomerular filtration rate according to local laboratory methods.

### 2.5. Follow-Up

All patients were followed up by the referring radiologist (H.V). Follow-up imaging was performed with either CT or gadolinium-enhanced MRI, with the first study being usually performed a minimum of 30 days post-procedure, then annually for 3 years. Then, a clinical follow-up was assessed every two years for four years, according to the patient’s choice. Clinical follow-up was provided by the referring interventional radiologist and at least once by the referent urologist. This angiographic follow-up examination was part of a continuous treatment strategy to show that embolization is successful in obliterating the angiogenic component and preventing bleeding and rupture. The efficacy of embolization was determined over a mean follow-up imaging period from embolization of 14.7 months (range: 0.06–57 months). Patients with incomplete follow-up imaging were contacted by telephone to verify the absence of recurrence and to ask for the most recent imaging study and laboratory test results. Recurrence was defined as an increase in tumor size on follow-up imaging, rebleeding, or recurrent symptoms requiring repeat embolization. Minor and major complications were defined according to the Society of Interventional Radiology Standards of Practice Committee guidelines [23]. Indications for repeat embolization included pain, hemorrhage, subsequent increase in tumor size, or persistence of intratumoral vascular pedicles.

### 2.6. Statistical Analysis

Quantitative variables were described as means and standard deviation (SD), medians, and ranges (min; max). Categorical variables were described by the number of participants and the associated percentage. Chi-2 test was used to compare proportions (or Fisher’s exact test if the expected frequencies are less than 5). For the study of tumor volume variation, paired tests were performed (Student’s test for paired series or Wilcoxon test for paired series based on normality). All statistical tests were two-sided, and *p* values < 0.05 were considered significant. Statistical analyses were performed with SAS Enterprise Guide version 8.2 software. Due to the low rate of missing values, we did not use any imputation method for the main outcome. Analyses were performed on complete cases.

## 3. Results

A total of 29 TAE were carried out in 24 patients, with a total of 25 AMLs treated at our center in Montpellier during the study period between June 2013 and March 2022.

### 3.1. Patients’ Characteristics

The mean age of the patients was 53.86 years (20–82 years old). The majority of patients were women (87.5%, n = 21 patients). One patient had an authenticated diagnosis of tuberous sclerosis complex, and the remaining patients had sporadic lesions with no underlying syndrome. Four patients (16%) underwent emergency embolization for active bleeding after stabilization, including one hypovolemic shock. The remaining were embolized electively (n = 13 patients, 52%), in which eight patients (32%) had symptoms associated with their AML: two patients had isolated hematuria, four patients had isolated flank pain, and two patients had both. We found a significant statistical association (*p* < 0.05) between the presence of aneurysms at diagnosis and the symptomatological nature of AML. Table 1 presents the main patient characteristics.

### 3.2. Angiomyolipoma Characteristics

The average arterial pedicle per AML was 1.4 (±0.8). Arterial pedicle count was the number of angiographically visible arterial pedicles that were a direct division of the renal artery and participated in the vascularization of the tumor. A high arterial pedicle count was significantly predictive of a second embolization (*p* < 0.01). The mean pre-embolization volume was 201.1 (±504.1) mm^3^. The majority of angiomyolipomas were exophytic (96%, n = 24). Ten patients had aneurysms on angiography (Figure 2a and Figure 3), including only five seen on pre-embolization imaging at diagnosis (Figure 1). Table 2 presents the different AML characteristics.

### 3.3. Selective Arterial Embolization and Volume Shrinkage

**Figure 4:**
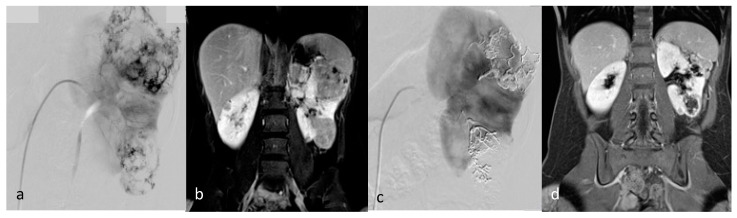
Selective arterial embolization (TAE) of two large left AML. (**a**) Angiography shows two AML with dysplasic vascularization. (**b**) MRI showing the two AML on coronal T1 FatSat VIBE Sequence with Gadolinium with hyposignal T1 aneurysms before TAE. (**c**) Final angiographic control after TAE of the two arterial branches with ONYX 18 (6% EVOH) with technical success (lack of opacification of the AML) (**d**) MRI showing the two AML on coronal T1 FatSat VIBE Sequence with Gadolinium after TAE, with a volume shrinkage of 83 % for the upper one and 49% for the lower one.

The therapeutic indication for Onyx embolization was based on size in 92% of cases (n = 23 patients), on symptomatology in 32% of patients (n = 8 patients), on bleeding in 16% of them (n = 4 patients), and in 20% of patients on the presence of an aneurysm (n = 5 patients). The mean post-embolization volume was 93.4 (±253.1) cm^3^, with a mean volume reduction of 107.7 cm^3^ (±267.3) per lesion or 53.6% (±28.3%) (Figure 4).

We had a total technical success of 96% with a primary technical success of 88% after only one embolization (23 AMLs) and a secondary technical success of 92% after two embolization (2 AMLs). Two patients had a scheduled embolization in two times because of the multitude of vascular pedicles, in order to limit the duration of the procedure. Two other patients presented a partial revascularization of their AML after 274 days and 547 days respectively, by new vascular pedicles after a first successful embolization. Their second embolizations were complete without recurrence. A total of four AMLs had two embolizations.

**Figure 5:**
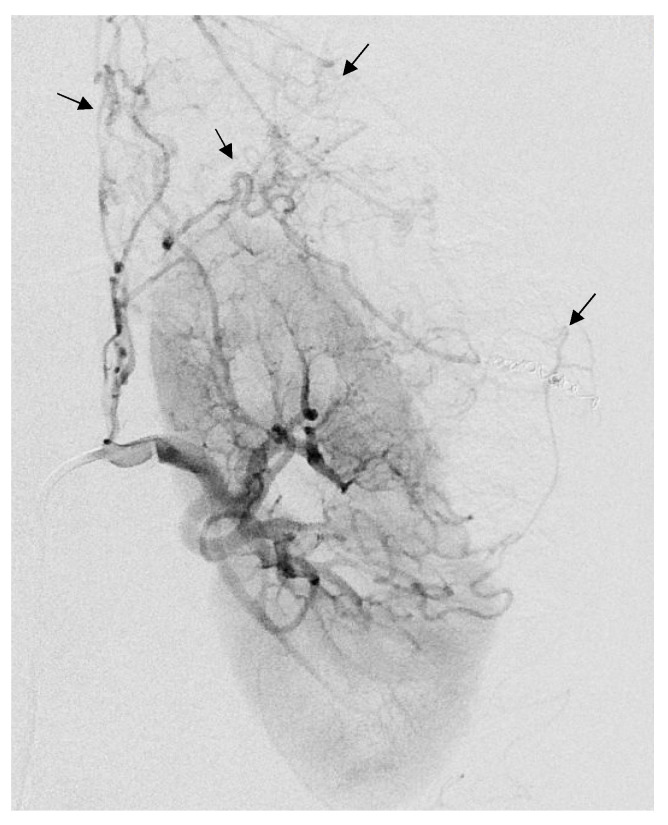
Arteriogram showing a large left AML with anarchic vascularization (black arrows), as part of a Tuberous sclerosis complex in a 32-year-old patient.

Three patients had been embolized with other embolization agents before Onyx. One patient, with tuberous sclerosis complex (Figure 5), had three previous embolization with microparticles (700 and 900 microns) and supplemental coil occlusion before embolization with Onyx. One other patient had a previous embolization with 900-micron microparticles. Finally, another patient had been embolized with two micro-coils in another center.

The mean amount of Onyx per embolization was 1.2 mL (±2.3). The average amount of Onyx used per patient was 1.4 (±2.4) mL. Three AMLs were embolized with Onyx 34 (12%) and 22 with Onyx-18 (88%). Two patients had post-embolization surgery (8%)**.** After a multi-disciplinary conciliation meeting, five embolizations, including two with EVOH and an AML regrowth by recruitment of neo-vessels, one patient had a total nephrectomy in the context of tuberous sclerosis complex. Subsequently, the patient also had active bleeding within the nephrectomy lodge. Concerning the other patient, after a first embolization, there was a development of tumor neovascularization resistant to embolization from suprarenal, lumbar, and diaphragmatic arterial branches, which was not safe to embolize considering the high risk of non-targeted embolization. An indication of left enlarged nephrectomy by subcostal laparotomy was therefore given after a multi-disciplinary conciliation meeting. A total of 80% of our patients (n = 20) had general anesthesia versus 20% with hypnosedation (n = 5). The average dose of contrast injected by embolization was 53.5 (±25.4) mL. The average dosimetry per embolization was 55.1 (±52.1) Gy.cm^2^.

### 3.4. Other Outcomes

Follow-up time, defined as the time from the first embolization to the last available imaging, was 448,6 (2–1750) days on average. None of the patients developed significant changes in renal function following embolization. The recurrence rate was 4% and concerned only one patient with tuberous sclerosis complex for whom surgery had to be considered. There was no recurrence concerning sporadic AML. There was no rebleeding during the post-embolization follow-up of our patients. Pre-therapeutic imaging was mostly CT (60%, n = 15) versus 40% by MRI (10 patients). Post-therapeutic imaging was mostly performed by MRI (76%, n = 19) rather than CT (24%, n = 6) because of artifacts related to Onyx. None of the patients presented with acute bleeding or pain during follow-up. Disease-specific survival of the entire cohort was 100%. Two patients had a tumor volume reduction of less than 5%. One of them had a radiological follow-up for only 2 days. The patient did not wish to continue the radiological follow-up but has been asymptomatic for almost 2 years now (no bleeding, no symptoms). The initial presentation was a hemorrhagic AML. The other patient had a 98-day follow-up with high-fat content AML. No rebleeding nor new symptoms were found in this patient.

### 3.5. Complications

**Figure 6:**
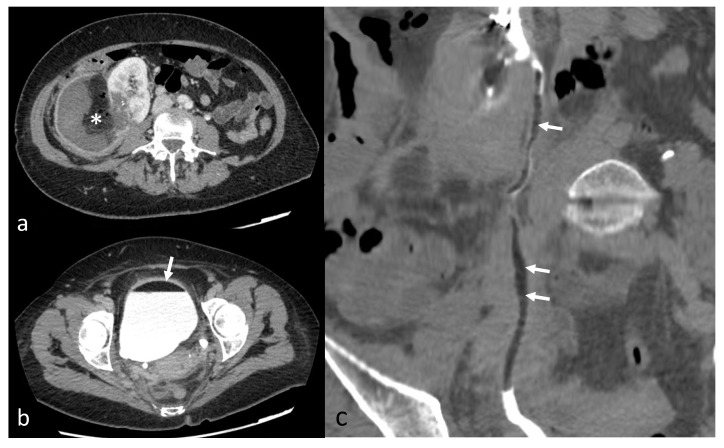
**Postembolization lipuria in a 70-year-old patient with a right AML**. (**a**) Intratumoral necrosis (*) on axial computed tomography with injection and urinary acquisition (**b**) Fat-supernatant indicating lipuria (white arrow) on axial computed tomography with injection and urinary acquisition (**c**) Intra-ureteral fat (white arrows) on coronal computed tomography with injection and urinary acquisition.

Minor complications were recorded in three patients (12%) and major complications in two patients (8%). One patient had a non-targeted embolization of the renal parenchyma during embolization (minor complication). The Onyx was not correctly visible (on poor homogenization with tantalum powder). The non-target embolization represented 28% of renal parenchyma, with no alteration of renal function at follow-up. One other patient had a pseudoaneurysm of the punctured femoral artery resulting in an additional day of hospitalization (minor complication). Doppler follow-up showed the resolution of the latter after being successfully treated by ultrasound-guided compression and monitoring. One patient had a post-embolization syndrome (PES) 48 h after embolization, with further anti-inflammatory and analgesic treatment (minor complication). One patient had a renal artery dissection during embolization, ultimately leading to non-target embolization of 20% of the kidney (major complication). The patient then had a pulmonary embolism and the appearance of a bilateral pleural effusion. She presented with hypovolemic shock due to a massive retroperitoneal rupture of the tumor. She also developed healthcare-associated pyelonephritis (major complication). One other patient presented, 15 days after embolization, a superinfection of the embolization site with fistulization in the pyelocaliceal system, leading to lipuria (Figure 6). The patient was hospitalized with antibiotics and analgesics (major complication). The AML drained itself almost completely into the pyelocaliceal cavities, conducting a volume shrinkage of 99%.

## 4. Discussion

Our retrospective single-center study of TAE with EVOH shows its efficacy and safety in the management of AML to prevent hemorrhagic risk. It has a high success rate with few complications. To our knowledge, this study is the largest cohort study (n = 24 patients) concerning TAE using EVOH with 25 AML and 29 embolizations. Moreover, it is the first study about AML’s TAE using EVOH to evaluate tumor shrinkage with tumor volume in the literature. The tumor shrinkage was significant, and no patient experienced any effect on their renal function after TAE, neither rebleeding nor rupture during follow-up. We have shown that TAE of AML with Onyx results in an average volume reduction by half.

AMLs are benign tumors whose main risk is bleeding due to their dysplastic vascular components. Their hemorrhagic risk can lead to a more dramatic complication, with tumor rupture and retroperitoneal hemorrhage, called “Wunderlich syndrome [13,24]”, and can be life-threatening. In addition, given their benign nature, treatments that preserve the renal parenchyma should be preferred to avoid renal function impairment. TAE, introduced in the 1980s, is an interesting treatment with low complication rates [14], which targets tumor vessels. By obstructing the dysplastic tumor vasculature and aneurysms, it limits the risk of bleeding and stops the tumor growth, which even becomes hypertrophic, also allowing the management of symptomatic AML due to their size. In the case of multiple lesions, notably in the tuberous sclerosis complex, TAE is a favored alternative because it is superselective and allows sparing of the normal renal parenchyma, targeting only the tumor vasculature. Such a decision was made after a multi-disciplinary conciliation meeting and surgery consultation, especially for AMLs > 4 cm. The surgical indication should be reserved for TAE failures, for cases where embolization is too complex, or if malignant evolution is suspected (e.g., intratumoral necrosis, calcifications) [25]. There is currently no consensus on the preferred use of an embolization agent in the management of AML, and no study has yet demonstrated the superiority of one. Coils and microparticles are permanent embolic agents that induce clot formation but are, therefore, dependent on a normal coagulation status with an increased risk of clinical failure with patients presenting coagulopathy [26]. Many patients in hemorrhagic shock have, indeed, an acquired coagulopathy [26] that may limit their effectiveness. In addition, coils have a very proximal occlusion level and particles have a very distal occlusion level (capillary bed), similar to ethanol. Their isolated use favors the emergence of collaterals and neovascularization and, thus, leads to tumor regrowth [27]. N-butyl cyanoacrylate glue, for its part, depend upon substantial experience (high learning curve) because of its quick polymerization, requiring rapid and optimal control of the injection by an experienced operator to avoid unpredictable penetration [6]. On the other hand, Onyx, due to its properties, does not depend on coagulation to be effective, acts at various vascular levels, and polymerizes slowly with a controllable injection. It was historically used in neurovascular malformations [19] and has become popular for peripheral applications as a novel embolization agent. Due to its viscous, lava-like nature and slow polymerization, it allows precise control of embolization to reduce the risk of off-target embolization and infarction of healthy parenchyma [20]. Its non-absorption in blood makes the embolization definitive as it remains in the vessel, unlike ethanol, for example. The progression of the EVOH is made possible by the pressure applied on the injection syringe by the operator and not by blood flow. This ensures a very safe procedure, allowing the flow to be stopped instantly. The risk of reflux is, therefore, very limited if the injection is performed slowly, allowing embolization of short arterial pedicles, which is usually counter-indicated in the use of cyanoacrylates for example. In addition, Onyx has a less extravascular inflammatory effect compared with cyanoacrylates, resulting in a more tolerable experience for patients [6,19].

However, AML could potentially grow. Although benign neoplasms, their size increase leads to an enhanced risk of rupture or being symptomatic [28]. A 4 cm cut-off was historically used to indicate active treatment of AML, according to Oesterling et al. in 1986 [5] and various studies [5,29]. Nevertheless, this criterion has recently been questioned by several studies. Yamakado et al. [2] showed that this criterion had a sensitivity and specificity of 100% and 38%, respectively, to predict an AML’s rupture. Rimon et al. [30] have shown that large AMLs with small vascular composition are less likely to bleed. We used this historical criterion but associated it with other criteria such as the presence of aneurysms > 5 mm, symptomatology, or on patient request. Some centers added other criteria of active treatment for AML: age, rate of growth, women planning pregnancy, patients requiring prolonged anticoagulation, and patients with no rapid access to an appropriate medical care facility [4,31]. We found a significant statistical association (*p* < 0.05) between the presence of aneurysms at diagnosis and the symptomatological nature of AML, suggesting that dysplasic vasculature tend to be symptomatic as tumor growth, consistent with the pathophysiological data available on AML in the literature.

Post-embolization tumor size reduction with Onyx was significant in our cohort, in agreement with values already presented in the literature. Two patients had a tumor volume reduction of less than 5%. The first patient had a radiological follow-up of only 2 days and did not wish to continue the subsequent radiological follow-up. The patient has been contacted and had no symptoms or rebleeding 2 years after her embolization of an initially hemorrhagic AML, with optimal clinical success. We, therefore, decided to keep this patient in our study since embolization was successful on the hemorrhagic risk and the symptomatology. The other patient had a 98-day follow-up with high-fat content AML. No rebleeding nor new symptoms were found in this patient. We suppose that this minor post-embolization size reduction was due to this high-fat content. Indeed, several studies [6,32] showed that a low-fat content (<50%) predicted greater volume reduction (*p* < 0.001) since TAE has a greater effect on highly vascularized components of AML. Thus, because embolization is effective on the hypervascular portion of the AML responsible for the risk of bleeding, the measurement of the decrease in volume of AML may not be the best criterion for evaluating the effectiveness of embolization, especially in high-fat content tumors, which are less vascularized. Reduction of the non-fat component may be a more relevant criterion. Neither myoid nor vascular components should enhance on postcontrast imaging after TAE [27], and pre/postcontrast MRI is probably the ideal modality. CT is not recommended because the tantalum powder contained within the Onyx (to aid fluoroscopic visualization) causes streak artifacts. A large majority of our cohort patients were indeed followed by MRI after TAE. Standardized MRI follow-up could be suggested to optimize the analysis of post-embolization AML in our center.

We had three minor complications in our cohort, including only one post-embolization syndrome (PES). It is an inflammatory reaction caused by arterial embolization (regardless of the embolizing agent used), inducing fever and pain within 48 h of embolization. Its occurrence in our cohort was particularly low, with only one patient concerned, compared to other series with preventive PES treatment. Lin et al. [33] showed in a meta-analysis of 653 patients, with 54% of PES, in 30 studies. This low rate is possibly related to the low inflammatory nature of Onyx. However, Urbano et al. [34]. reported an 18.5% rate of PES in AML embolization with Onyx, supporting the idea that, in our study, anti-inflammatory premedication plays a major role in PES’ low incidence.

We had three major complications concerning two patients in our study. One patient had a renal artery dissection during embolization, ultimately leading to non-target embolization of 20% of the kidney. In the context of her hemorrhagic shock, there was a prior diffuse vasospasm of the visceral arteries, making navigation of endovascular catheters more delicate and more likely to cause endothelial damage. The patient then developed healthcare-associated pyelonephritis. The presence of a voluminous post-hemorrhagic perirenal hematoma, which was compressing the ureter, was probably preventing urine from draining properly and promoting a local infection. Superinfection of large hematomas is also common in intensive care units. She also had a pulmonary embolism after embolization and the appearance of a bilateral pleural effusion. Transfusion is known to be associated with increased thromboembolic risk due to increased blood viscosity with new red blood cells. Massive vascular filling during shock states is also known to favor the development of pleural effusions. She was discharged from the hospital with no deterioration of her renal function or her quality of life on follow-up. One other patient had liquefaction of her AML with fistulization in the pyelocaliceal system, draining itself into the urinary tract. Liquefaction of the AML after TAE is secondary to ischemic necrosis and occurs regardless of the embolization agent used [6].

Two patients underwent nephrectomy after TAE with Onyx (8%), as found in literature [3]. One had a tuberous sclerosis complex with multiple AML regrowth and numerous embolizations with coils, microparticles, and Onyx. Nephrectomy was assessed after a multi-disciplinary conciliation meeting, and it was the only case of tumor regrowth in our cohort. The surgical report describes a hemorrhagic kidney at the slightest manipulation with no visualization of the renal artery. Kuusk et al. [35] found that TAE is generally less effective than partial nephrectomy for tumors larger than 8 cm, in agreement with the study of Chan et al. [14] with AML larger than 10 cm. Their anarchic vascularity makes embolization more complex and less effective, with usually recurrent TAE [36]. Kuusk et al. [35] showed that 17% of tuberous sclerosis complex-associated AML demonstrated at least 20% of growth after an initial decrease in volume. Nevertheless, AML associated with tuberous sclerosis complex are frequently bilateral, and a systematic nephrectomy would result in a significant loss of functional renal tissue, leaving embolization room for first-line treatment. Concerning the other patient who underwent nephrectomy, the appearance of tumor neovascularization after TAE from suprarenal, lumbar, and diaphragmatic arterial branches was not safe to embolize, considering the high risk of non-targeted embolization. The risk of non-targeted ischemic lesions should be kept in mind when treating a tumor with multiple feeding arteries. As explained by Lee et al. [25], our goal was to preserve renal function as much as possible while limiting the risk of tumor bleeding. This is consistent with the fact that the number of arterial pedicles was significantly associated with a secondary embolization in our cohort study.

Moreover, TAE with EVOH has some limitations: the need for compatible microcatheters, the use of DMSO, and the proper costs of the device mainly [17]. DMSO solvent is obligatory to keep EVOH dissolved before injection, and it can cause endothelial necrosis, pain, and vasospasm in the event of quick injection [17,18]. The usual doses injected during embolization are well below the toxic dose of DMSO. However, it should always be injected slowly and in a controlled manner to avoid vasospasm and endothelial necrosis. EVOH also raises the local temperature and can generate acute burning pain. That is why most of our patients underwent general anesthesia, and only a few had hypnosedation in emergency circumstances. General anesthesia is also required because it allows strict immobility for a precise and usually long procedure. Moreover, the tantalum powder radiopacity may interfere with post-operative CT scan imaging but is counteracted by the use of MRI follow-up. Finally, some authors highlight the high cost of Onyx as a potential limiting factor considering that compatible catheters are also more expensive than their more common counterparts [17,24].

Several limitations should be considered when interpreting the results of our study. The main limitation is its retrospective nature and relatively small cohort size. We only had one patient with tuberous sclerosis complex over 24 patients, while they represent 20% of patients with AML in the general population [1]. This is explained by a very low incidence of tuberous sclerosis complex in the general population (about 0.01% [37]). The most common Onyx concentration used was 6% (Onyx-18), not allowing for the study of a possible link between Onyx concentration and AML size reduction or complications. Moreover, tumor volume was calculated using orthogonal measurements as a gold standard, which assumes that the masses are ellipsoid. However, AMLs have various shapes and could be asymmetrical [13], leading to possible inadequate volume estimation. Some centers used a standardized, validated software program [6] for volumetric analyses. Furthermore, active treatment was chosen at the discretion of the treating urologist and interventional radiologist rather than by a defined prospective protocol. The mean follow-up was rather short (442 days, 14.5 months) and varied considerably (2–1750 days). Patatas et al. [38] showed that the main volume reduction after TAE is attained in the first year, suggesting a minimum standardized follow-up of one year after embolization, in accordance with our results. Finally, other liquid embolization agents are now available with complementary characteristics to ONYX, such as SQUID [39,40] or PHIL [41,42]. SQUID has the same components as ONYX but with smaller tantalum grains, allowing for better homogeneity of the embolizing agent under fluoroscopy and longer duration during long procedures [43]. PHIL, a completely different polymer, has similar characteristics but does not require agitation before use [43]. Further studies comparing these different agents would be useful, particularly in the embolization of angiomyolipoma. However, with AML being a rare tumor, conducting such studies would be complex to implement.

## 5. Conclusions

In conclusion, this article provides an evidence-based approach to support the treatment of AML with TAE using EVOH, in accordance with the literature, targeting tumor vasculature to avoid AML’s main complication: hemorrhage. TAE with Onyx is feasible, effective, and safe. It is well tolerated by patients and associated with minimal adverse events allowing the preservation of renal function. Moreover, as a minimally invasive technique, recurrent TAE is feasible in daily practice.

## Figures and Tables

**Table 1:** Mean features of the 24 study patients (25 AML).

Variable	Value
N. of patients	24
N. of embolized AML	25
N. of embolization	29
Females/Males, n (%)	21 (87.5)/3 (12.5)
Age (years), mean ± SD	53.9 (±16.2)
tuberous sclerosis complex n (%)	1 (4)
Clinical Presentation, n (%)	
Symptomatic	8 (32)
Emergency	4 (16)
Incidental discovery	13 (52)
Therapeutic indication	
Size ^a^	23 (92)
Bleeding	4 (16)
Aneurysm ^b^	5 (20)
Pre-therapeutic imaging: CT/MRI, n (%)	15 (60)/10 (40)
Post-therapeutic imaging: CT/MRI, n (%)	6 (24)/19 (76)
Side of AML: left/right, n (%)	14 (56)/11 (44)
Location: Exophytic/Intraparenchymatous, n (%)	24 (96)/1(4)
Previous embolic agents, n (%)	3 (12)

AML, renal angiomyolipoma; n, number; ^a^ tumors > 4 cm in size; ^b^ aneurysm > 5 mm in size.

**Table 2:** Outcomes, Selective arterial embolization (TAE) features, follow-up, kidney function, and tumor size before and after TAE.

Variable	Value	
	Mean (±SD)	Median (Range)
N. of arterial pedicles per AML	1.4 (±0.8)	1 (1–4)
AML before TAE		
Size, cm	70.9 (±38.0)	54 (41–203)
Volume, cm^3^	201.1 (±504.1)	41.47 (13.2–2539.8)
Radiologic follow-up, days	448.6 (±473.8)	248 (2–1735)
AML after TAE		
Size, cm	53.1 (±34.1)	48 (21–169)
Volume, cm^3^	93.4 (±253.1)	24.92 (2.4–1223.1)
Tumor size reduction		
cm	17.78 (±21.1)	12.4 (−2–98.5)
%	24.38 (±20.3)	18 (−1.4–77.9)
Tumor volume reduction		
cm^3^	107.7 (±267.3)	25.3 (−14.3–1315.8)
%	53.6 (±28.3)	51.8 (−3.2–99.1)
Volume reduction < 5%	2 (8)	
Size increasing after TAE, n (%)	1 (4)	
Onyx (EVOH)		
Type used: 18/34, n (%)	22 (88)/3 (12)	
Quantity per TAE, mL	1.2 (±2.3)	0.5 (0.1–11)
Total quantity used per patient	1.4 (±2.4)	0.6 (0.2–11)
Serum creatinine level		
Before TAE, μmol/L	70.5 (±24.2)	63 (49–161)
After TAE, μmol/L	68.3 (±17)	64 (49–122)
Technical success rate, n (%)	24 (96)	
After one embolization	23 (92)	
After two embolizations	1 (4)	
N. of TAE per AML	1.2 (±0.4)	1 (1–2)
Need for re-embolization, n (%)	4 (16)	
Renal surgery after TAE, n (%)	2 (8)	
Minor complications after TAE, n (%)	3 (12)	
Non-targeted embolization	1 (4)	
Femoral pseudoaneurysm	1 (4)	
Post-embolization syndrome	1 (4)	
Major complications after TAE, n (%)	3 (8)	
Abcess with lipuria	1 (4)	
Arterial Dissection	1 (4)	
Healthcare associated pyelonephritis	1 (4)	
Anesthesia: General/Hypnosedation, n (%)	20 (80)/5 (20)	
Rebleeding after TAE, n (%)	0	
Recurrence, n (%)	1 (4)	
Tumor regrowth	1 (4)	
Dosimetry per TAE: Gy.cm^2^, n (%)	55.1 (± 52.1)	31.0 (6.9–215.2)
Contrast agent used per TAE: mL, n (%)	53.5 (±25.4)	46 (10–100)

AML, renal angiomyolipoma; *n*, number; TAE, transarterial arterial embolization.

## Data Availability

All the study data are reported in this article.
